# Protein intake and injury outcomes among fallers in the Women’s Health Initiative’s Objective Physical Activity and Cardiovascular Health in Older Women Study

**DOI:** 10.1371/journal.pone.0353769

**Published:** 2026-07-22

**Authors:** Shelby G. Ziller, Leslie K. Dennis, Jeannette M. Beasley, Andrea Z. LaCroix, Jean Wactawski-Wende, Linda Snetselaar, Jane A. Cauley, Bernhard Haring, Abdul Tawab Saljuqi, Jennifer W. Bea

**Affiliations:** 1 Department of Epidemiology and Biostatistics, The University of Arizona, Tucson, Arizona, United States of America; 2 Departments of Nutrition and Food Studies and Medicine, New York University, New York, New York, United States of America; 3 University of California San Diego, Herbert Wertheim School of Public Health and Human Longevity Science, San Diego, California, United States of America; 4 Department of Epidemiology and Environmental Health, University at Buffalo, State University of New York, Buffalo, New York, United States of America; 5 Department of Epidemiology, University of Iowa, Iowa City, Iowa, United States of America; 6 Department of Epidemiology, University of Pittsburgh, School of Public Health, Pittsburgh, Pennsylvania, United States of America; 7 Department of Epidemiology and Population Health, Albert Einstein College of Medicine, Bronx, New York, United States of America; 8 HOMICAREM (HOMburg Institute of CArdioREnalMetabolic Medicine), Saarland University, Homburg, Germany; 9 Department of Medicine IV, Clinic Hietzing, Vienna Healthcare Group, Vienna, Austria; 10 Department of Surgery, The University of Arizona, Tucson, Arizona, United States of America; 11 Department of Health Promotion Sciences, The University of Arizona, Tucson, Arizona, United States of America; Dynamical Business & Science Society - DBSS International SAS, COLOMBIA

## Abstract

**Background:**

Falls among older adults are associated with increased morbidity, mortality, and healthcare costs; therefore, it is critical to assess modifiable risk factors. This study aimed to determine if low protein intake among older women was associated with injuries among fallers.

**Methods:**

The Women’s Health Initiative ancillary studies, Live Long Study (LLS), Food Intake (FI), and Objective Physical Activity and Cardiovascular Health in Older Women study (OPACH) were combined for analyses (n = 6,580; n = 1,285 fallers). Protein intake was assessed by food frequency questionnaires at LLS/FI/OPACH baseline. Participants completed fall calendars for 13 months and self-reported injuries in interviews. Logistic regression determined odds ratios (OR) and 95% confidence intervals (CI) for risk of injurious falls and falls with fracture. Diabetes medication use had a significant interaction between protein density and fall with any injury; thus, models were stratified.

**Results:**

Of the participants who fell, 841 (65.4%) fell without injury, 444 (34.6%) fell with injury, and 70 (7.7%) fell with fracture. Lower protein density (<15% calories from protein) increased risk of fracture from falling (OR: 1.73; 95% CI: 1.02–2.92) compared to those with higher protein density (≥15% calories from protein). Among participants with diabetes medication use and lower protein density, the OR for any injury from falling was 5.54 (95% CI: 1.79–17.19) compared to those with higher protein density though based on a small subgroup.

**Conclusion:**

Lower protein density increased risk of injurious falls. Dietary and medication screening may facilitate selection and tailoring for behavioral falls prevention to reduce injurious falls and fractures.

## Introduction

Every year, over one-quarter of community-dwelling adults aged 65 or older fall [1]. Around half of those falls result in an injury, with 10% of those injuries being severe [2]. Individuals 65 years or older who fall are twice as likely to fall again [3]. As the number of adults in this at-risk age group increases with the incoming generation, falls will become a greater burden on communities and the healthcare system [4]. Consequently, identifying and mitigating fall risk factors has become increasingly crucial, given established falls associations with morbidity, mortality, and healthcare expenditure [1,2,5].

Falls risks have traditionally been categorized by intrinsic or extrinsic factors [2]. Intrinsic factors, such as age, chronic illnesses, and cognitive impairment, are inherent to the individual. However, extrinsic factors, such as diet quality, medication usage, footwear, and alcohol/drug consumption, are modifiable and present opportunities for intervention in this population [2]. Recent studies have shed light on the potential role of dietary protein in mitigating falls risk. Lower protein intake has been associated with increased risks of falling, fractures, frailty, and skeletal muscle loss in specific populations, including older women in the Women’s Health Initiative (WHI) [6–12]. However, while research has examined protein intake with the risk of falling, fractures, and fall-related measures, examining the risk of injury and fracture among those who fall is unexamined in the WHI and much of the literature at large [6,7,9,12–17]. These associations raise the intriguing hypothesis that protein intake could serve as a modifiable risk factor for injury outcomes from falls among older adult fallers in the WHI.

The primary aims of this study were to determine if low protein intake was associated with an increased risk of 1) a reported injury outcome among those who fell and 2) a reported fracture from falling among those who fell. Additionally, the secondary aims were to examine whether low protein intake was associated with 1) an increased risk of treatment sought after falling with any reported injury outcome and 2) an increased risk of falling with a fracture among those who reported any other injury from a fall. Finally, the sensitivity analyses will assess animal and vegetable protein separately for their associations with 1) a reported injury outcome among those who fell and 2) a reported fracture from falling among those who fell.

## Materials and methods

### Study population

This study examined the risk of falling with an injury among fallers in the WHI companion ancillary studies, the Live Long Study (LLS), Food Intake (FI) study, and the Objective Physical Activity and Cardiovascular Health in Older Women (OPACH) study. The main WHI and the LLS/FI/OPACH ancillary studies have been previously described [18–21]. In brief, the WHI is a prospective cohort of 161,808 postmenopausal women in the US that began recruitment from Sept 1993 to Dec 1998 and ran the initial study until 2005 [18,19]. The study has since been extended, and follow-up continues annually.

The LLS was an ancillary study of the WHI (n = 7,875) from March 2012 to May 2013 and consisted of older women from the main WHI [20,21]. From late 2011 to mid-2012 women were recruited to the LLS study with surviving African American/Black and Hispanic women of the WHI being prioritized for participation for an improved distribution of race and ethnicity in comparison to the WHI [20,21]. During LLS, participants completed a baseline questionnaire and an at-home visit where physical ability tests and anthropometrics were assessed [20]. The FI study then mailed out a food frequency questionnaire (FFQ) in March 2012 to May 2013 to all LLS participants, and 6,095 were returned [20]. OPACH was a follow-up study of the LLS participants from March 2012 to March 2015 (n = 7,048) [21]. During OPACH, 6,580 participants were enrolled in the falls cohort, and 5,776 completed at least one month of the 13-month fall count follow-up [21]. The WHI project was reviewed and approved by the Fred Hutchinson Cancer Research Center (Fred Hutch) IRB in accordance with the U.S. Department of Health and Human Services regulations at 45 CFR 46 (approval number: IR# 3467-EXT). Participants provided written informed consent to participate. Additional consent to review medical records was obtained through signed written consent. Fred Hutch has an approved FWA on file with the Office for Human Research Protections (OHRP) under assurance number 0001920.

**Fig 1:**
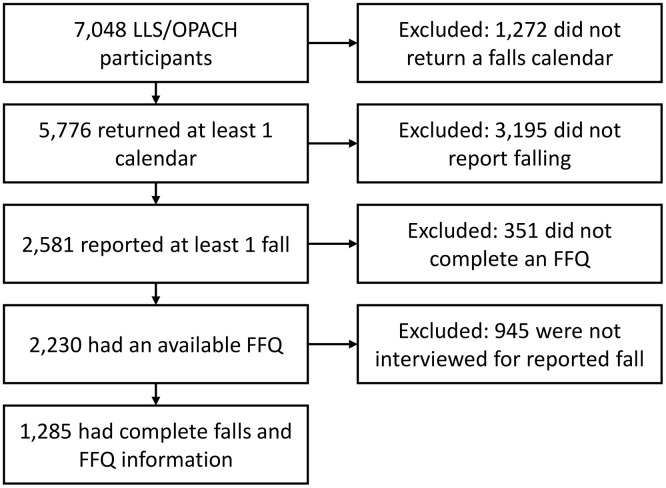
Sample Size and Eligibility Criteria for LLS/FI/OPACH participants. Abbreviations: LLS: Live Long Study; FI: Food Intake Study; OPACH: Objective physical activity and cardiovascular health study; FFQ: Food Frequency Questionnaire.

The LLS/FI/OPACH combined study had 6,580 participants (Fig 1). LLS/FI/OPACH participants were eligible for the present analyses if they have 1) completed the FI FFQ, 2) returned at least one fall calendar with a recorded fall, and 3) completed at least one fall interview (Fig 1). Participants with a recorded fall but no interview were excluded due to the lack of information on injury outcomes. Fall interviews were completed for as many reported falls as possible within the study’s resources [21], therefore, only a participant’s first fall interview was included in the analysis.

### Protein intake

FI researchers utilized the General Nutrition Assessment (GNA) FFQ to collect dietary intakes [20]. The FFQ consisted of 125 items asking about frequency and consumption over the past three months [20]. The Nutrition Data System for Research software version 2012 was then used for nutrition calculations [20]. The exposure of interest was protein intake taken from the GNA FFQ completed at FI enrollment [20]. Total protein intake in the GNA FFQs was created from the sum of all animal and vegetable protein in a day [20]. Total protein intake was then utilized to develop the primary exposures. The first exposure was low protein by weight, defined as whether participants had met their recommended dietary allotment (RDA) of protein: 0.8g protein per kg of body weight (eq. 1) [10].


$$\begin{array}{c} \text{0}.\text{8}\;\times body\;weight\;(kg)=protein\;cutoff\;per\;participant \end{array}$$ (1)

If participants met their cutoff point, they were defined as normal protein; if they did not, they were defined as low protein. In sensitivity analyses, we additionally tested 1.0g protein per kg of body weight as the participants cutoff. Additional cutoff points for 1.2, 1.4, and 1.6 g of protein were tested, but are not presented due to insignificant findings.

The second exposure was lower protein density, defined as whether participants had at least 15% of their daily calories from protein (eq. 2).


$$\begin{array}{c} \frac{Total\;protein\;(g)\;\times \text{4}}{Total\;calories\;(K\;cal)}\;\times \text{1}\text{0}\text{0} \end{array}$$ (2)

Participants with 15% or higher protein density were defined as having higher protein, whereas those below 15% had lower protein [13]. The Acceptable Macronutrient Distribution Range (AMDR) recommends that 10–35% of total calories should come from a protein source [22,23]. In our study, we chose 15% as the lower protein density cutoff, rather than 10%, because only 20 (1.56%) participants had less than 10% protein density.

Sensitivity analysis used total animal protein and total vegetable protein values from the FFQs. In both analyses, the median value of the protein source was used as the cutoff point between high and low protein levels, due to the lack of recognized cutoff points in the literature. Animal protein was cutoff at 37.72 g, and vegetable protein was cutoff at 21.08 g.

### Falls and fractures

During OPACH follow-up, participants recorded on calendars every day if they had or had not fallen and returned the calendars to researchers at the end of each month. Women who recorded a fall were interviewed about the details of their fall, including detailed information about injury outcomes, location, and treatment sought [21]. Injury status and treatment sought were self-reported only and not adjudicated with medical records. Using the fall interviews, two indicators of injury outcomes were created for the primary aims. The first outcome was falling with injury outcome among fallers, dichotomized into women who fell with no reported injury and women who fell with any reported injury. In the second outcome, injuries were limited to fractures, and the outcome was dichotomized into women who fell with no reported injury and women who fell with self-reported fracture. In secondary analyses, the treatment of fall related injuries among fallers with an injury was included. The outcome was the binary variable: fall with untreated or treated injury from a fall. Treatment was divided by the level of treatment sought out. If an individual received treatment at a doctor’s office, went to the ER, or was admitted to the hospital, they were recorded as injured with treatment. An individual who treated themselves or was treated outside the medical setting was deemed injured without treatment. In an additional sensitivity analysis, participants were limited to all those who reported an injury, and the outcome was dichotomized into women who reported a fracture and women who reported an injury but not a fracture. Further sensitivity analysis removed women who reported fractures without self-reported treatment inside a medical setting in both the primary and secondary analyses.

### Covariates

Baseline clinical and lifestyle characteristics of participants were included as covariates in the models. Age at OPACH baseline was taken from baseline OPACH questionnaires. Race and ethnicity were taken from the main WHI baseline. Height (to the nearest 0.5 inch) and weight (to the nearest pound) were measured by researchers during the LLS at-home assessment. BMI was then calculated as (weight (kg)/ height (m)^2^). Physical activity [metabolic equivalent of task hours/week (METs hrs/wk)] was assessed through validated self-report questionnaires [24]. Self-reported diabetes medication use (yes/no) was taken from baseline LLS questionnaires. Hormone therapy (HT) use is defined as ever or never user. This was determined through WHI baseline questionnaires, HT clinical trial arm membership, annual medicine use questionnaires, and the LLS baseline questionnaire. If participants had never taken a HT product based on all available information, they were deemed a never user; otherwise, they were categorized as an ever user. Physical ability tests included a grip strength test using a calibrated Jamar hand-grip dynamometer and the Short Physical Performance Battery (SPPB) of tests [25]. The SPPB consisted of balance, timed-walk, and gait tests with a maximum score of 4 available for each test, with total scores ranging from 0–12 [25,26]. Using the FI FFQ, researchers calculated the Healthy Eating Index (HEI) 2010 score. Membership in the clinical trial arms during the Main WHI (dietary modification trial, vitamin D and calcium trial, and HT trial) was also included as a covariate. Though not a selection parameter, in our smaller subsample of the LLS/FI/OPACH studies, the HT Trial members only included participants from the estrogen alone intervention and placebo arms.

### Statistical analysis

Descriptive statistics were stratified by total protein density status and reported means, standard deviations, percentages, and frequencies as appropriate. Chi-square tests were used to assess differences in protein and fall status. Analyses used logistic regression to determine the association between low protein and fall outcome. Interaction (p < 0.05) with protein was examined for age, falls history, self-reported vision, diabetes medication use, calcium supplementation, and HT usage. In the final models, age at OPACH baseline, race and ethnicity, and WHI randomization to clinical trial arms were controlled for based on a priori decisions. Confounding was determined by a change in the unadjusted odds ratio (OR) of 5% or greater for other risk factors. The cutoff was set to the more sensitive 5% as no potential confounders reached the traditional 10% cutoff point. This change from the traditional 10% is supported by evidence that the change-in-estimate thresholds likely vary based on study characteristics rather than a fixed universal values (i.e., 10%) [27]. Thus, the final models included age at OPACH baseline, race and ethnicity, BMI category (underweight, normal weight, overweight, obese), physical activity (METs hrs/wk), and clinical trial arm. Protein by weight, animal protein, and vegetable protein models were additionally adjusted for total calories. Protein density models did not include total calories, as it was used to calculate the protein density. Physical function assessed by grip strength or SPPB and the HEI 2010 score were not confounders in these associations and were therefore not included in the final models. However, the HEI 2010 score, grip strength, and SPPB were included in the descriptive statistics to illustrate diet quality and physical function.

Data were analyzed using SAS 9.4 (SAS Institute, Inc, Cary, NC). A type I error rate of 0.05 and 2-sided tests were used for all analyses.

## Results

After exclusions, 1,285 participants were included in the analyses (Fig 1). Of these participants, 841 (65.4%) fell without injury, while 444 (34.6%) fell with a reported injury, and 70 (7.7%) fell with a reported fracture (Table 1). Participants who were injured primarily were sore (74.5%) with bruising (65.5%) (S1 Table in S1 File). Fractures were reported across the entire body, with 28 (40.0%) in the lower extremities and 26 (37.1%) in the upper extremities (S2 Table in S1 File).

**Table 1:** Descriptive statistics by injury status for older women (n = 1285) in the Objective Physical Activity and Cardiovascular Health in Older Women (OPACH) study.

	Normal Protein Density	Lower Protein Density	Total	
	(N = 873)	(N = 412)	(N = 1,285)	
**Variables**	N (%)/ Mean (SD)	N (%)/ Mean (SD)	N (%)/ Mean (SD)	p-value
Age at OPACH baseline (yrs)	79.1 (6.6)	79.3 (6.3)	79.2 (6.5)	0.68
Race & Ethnicity*				
White	560 (64.3%)	241 (58.5%)	801 (62.4%)	0.03
Black/African American	166 (19.1%)	112 (27.2%)	278 (21.7%)	<0.01
Hispanic/Latina	129 (14.8%)	54 (13.1%)	183 (14.3%)	0.37
BMI (kg/m^2^)	26.8 (5.3)	26.2 (5.3)	26.6 (5.3)	0.98
BMI (kg/m^2^)*				
Normal weight (BMI 18.5–24.9)	347 (40.1%)	179 (43.6%)	526 (41.2%)	0.62
Overweight (BMI 25.0–29.9)	293 (33.8%)	135 (32.8%)	428 (33.5%)	
Obese (BMI ≥ 30.0)	204 (23.6%)	89 (21.7%)	293 (23.0%)	
Physical Activity (MET-hrs/wk)	15.1 (15.0)	12.5 (14.6)	14.3 (15.0)	<0.01
Ever use HT (yes)	433 (49.6%)	213 (51.7%)	646 (50.3%)	0.48
Diabetes medication use (yes)	76 (8.8%)	27 (6.6%)	103 (8.1%)	0.18
HEI 2010	73.6 (9.8)	68.1 (11.6)	71.8 (10.7)	<0.01
Total calories (kcal)	1,554.3 (646.3)	1,588.5 (751.7)	1,565.3 (681.8)	0.40
SPPB	8.3 (6.6)	8.1 (2.6)	8.2 (2.6)	0.27
Grip strength (kg)	17.4 (6.5)	18.6 (7.3)	17.8 (7.3)	0.01
HT Clinical trial member (yes)	613 (70.2%)	267 (64.8%)	880 (68.5%)	0.15
Estrogen alone intervention arm (yes)	305 (34.9%)	131 (31.8%)	436 (33.9%)	
CaD Clinical trial member (yes)	453 (51.9%)	222 (53.9%)	675 (52.5%)	0.72
CaD intervention arm (yes)	223 (25.5%)	110 (26.7%)	343 (26.7%)	
DM Clinical trial member (yes)	261 (29.9%)	134 (32.5%)	395 (30.7%)	0.43
DM intervention arm (yes)	103 (11.8%)	59 (32.5%)	162 (12.6%)	
Observation study member (yes)	167 (19.1%)	93 (22.6%)	260 (20.2%)	0.16
Total Protein (g)	70.6 (29.8)	52.2 (25.7)	64.7 (29.8)	<0.01
Low Total Protein (yes)	218 (25.0%)	236 (57.3%)	517 (40.2%)	<0.01
Total Vegetable Protein (g)	23.0 (10.9)	22.5 (12.2)	22.8 (11.3)	<0.01
Low Vegetable Protein (yes)	421 (48.2%)	222 (53.9%)	643 (50.0%)	0.06
Total Animal Protein (g)	47.6 (22.8)	29.7 (16.8)	41.9 (22.6)	<0.01
Low Animal Protein (yes)	323 (37.0%)	319 (77.4%)	642 (50.0%)	<0.01
Falls per month	0.2 (0.3)	0.3 (1.4)	0.3 (0.8)	<0.01
Fall with injury	291 (33.3%)	153 (37.1%)	444 (34.6%)	0.18
Fall with treated injury** (n = 418)	98 (35.3%)	51 (36.4%)	149 (35.6%)	0.81
Fall with fracture*** (n = 444)	41 (4.5%)	29 (3.2%)	70 (7.7%)	0.07

Note: In total, the OPACH study 2,219 (44%) women reported at least one fall, totaling 5,980 reported falls. Of those who reported a fall, 1,492 received an interview. *If a cell has fewer than 10 participants, the category was not reported in detail; therefore, some columns do not equal 100%. **Sample size includes only those who fell with injury and reported treatment. ***Sample size includes only those who fell with a reported injury. Falls per month are calculated as the total number of falls divided by the total number of calendars reported. Though not a selection parameter, in our smaller subsample of the LLS/FI/OPACH studies, the HT Trial members only included participants from the estrogen alone intervention and placebo arms. Abbreviations: BMI = Body mass index; MET-hrs/wk: the metabolic equivalent of task (hours/week), HEI: Healthy Eating Index 2010; HT: Hormone Therapy; CaD: Calcium and Vitamin D; DM: Dietary modification; LLS: Live Long Study; FI: Food Intake Study; OPACH: Objective Physical Activity and Cardiovascular Health in Older Women Study; SPPB: Short physical performance.

Overall, participants had a mean age of 79.2 years (±6.5), 14.3 (±15.0) physical activity hours/week (METs hrs/wk), 1563.3 Kcal (±681.8) for total calorie intake, 64.7g (±29.8) for total protein intake, where primarily white (62.4%), and normal weight (41.2%) (Table 1). Descriptive statistics of covariates stratified by protein density are further illustrated in Table 1. In the sensitivity outcome analyses examining treatment post-injury fall, 418 participants were included. Of those who fell with an injury, 149 (35.6%) sought treatment for their injury.

### Protein by weight

The majority of participants (59.8%) met the protein RDA based on their weight (<0.8g/kg weight). Of the women with low protein, 175 (33.9%) fell with an injury, whereas 296 (35.0%) of normal protein women fell with an injury (chi-square p = 0.66). Women who met their protein RDA had a mean total protein of 80.7g (±26.8), while women with low protein had a mean total protein of 41.0 g (±14.1). Of the women who fell with a fracture, 31 (8.3%) also had low protein by weight. Additionally, of women with normal protein 39 (7.2%) fell with a fracture (chi-square p = 0.55).

Participants with low protein by weight did not have significantly increased odds of falling with an injury or falling with a fracture compared to those meeting their RDA of protein by weight (Table 2). The majority of participants (60.1%) did not meet the protein by weight cutoff when it was changed to 1.0g protein/kg weight. Participants with low protein by weight (<1.0g/kg weight) also did not have significant findings with increased injurious fall or fall with fracture (S3 Table in S1 File).

**Table 2:** Associations between protein intake and fall by injury status (n = 1285) and protein and fracture from fall status (n = 911) in older women in the Objective Physical Activity and Cardiovascular Health in Older Women (OPACH) study.

	**Unadjusted**	**Adjusted**
	(n = 1285; 444 injured)	(n = 1241; 430 injured)
	OR (95% CI)	OR (95% CI)
**Injury Status**		
Protein by weight		
Normal protein	Ref	Ref
Low protein	0.95 (0.75, 1.20)	1.01 (0.73, 1.39)
	**Unadjusted**	**Adjusted**
	(n = 911; 70 fractures)	(n = 878; 67 fractures)
	OR (95% CI)	OR (95% CI)
**Fracture Status**		
Protein by weight		
Normal protein	Ref	Ref
Low protein	1.16 (0.71, 1.90)	1.39 (0.71, 2.73)

Normal protein = weight * 0.8 ≤ total FFQ protein; Low protein = weight * 0.8 > total FFQ protein; Injury status = all participants fell, outcome dichotomized into fell with and without any reported injury. A fall with injury was used as an event. Fracture status = all participants fell, outcome dichotomized into fell with and without any reported fracture. A fall with fracture was used as an event. Adjusted for total calories, age at baseline, BMI category, physical activity (MET-hrs/wk), race/ethnicity, and clinical trial arm membership. Abbreviations: BMI = Body mass index; MET-hrs/wk: the metabolic equivalent of task (hours/week); OR = Odds Ratio; CI = Confidence interval.

### Protein density

The majority of participants (67.9%) had normal protein density (Table 1). Of the women with lower protein density, 153 (37.1%) fell with an injury. Similarly, 291 (33.3%) fell with an injury among those with higher protein density (chi-square p = 0.66). Women with higher protein density had a mean total protein of 70.6g (±29.8), while women with lower protein density had a mean total protein of 52.2g (±25.7).

Any diabetes medication usage was found to modify the association of protein density and injurious fall (p = 0.01); thus, stratified models are presented (Table 3). The stratified results showed a significantly increased risk of falling with an injury and lower protein density, OR=5.11, among participants using diabetes medication (Table 3). Of the women who fell with a fracture, 29 (10.1%) also had lower protein density, and of women with higher protein density, 41 (6.6%) fell with a fracture (chi-square p = 0.07). In these models, diabetes medication use did not modify the association; therefore, stratified models were not presented. Women with lower protein density had a significantly increased odds of falling with a fracture of 1.73 compared to those who had higher protein density.

**Table 3:** Associations between protein density and fall by injury status (n = 1285) and protein and fracture from fall status (n = 911) in older women in the Objective Physical Activity and Cardiovascular Health in Older Women (OPACH) study.

	**Unadjusted**		**Adjusted**	
	**No diabetes** **medication use**	**Diabetes** **medication use**	**No diabetes** **medication use**	**Diabetes** **medication use**
	(n = 1169; 407 injured)	(n = 103; 37 injured)	(n = 1130; 396 injured)	(n = 98; 34 injured)
	OR (95% CI)	OR (95% CI)	OR (95% CI)	OR (95% CI)
**Injury Status**				
Protein density				
Higher protein density	Ref	Ref	Ref	Ref
Lower protein density	1.01 (0.83, 1.39)	**3.81 (1.52, 9.54)**	1.11 (0.85, 1.44)	**5.54 (1.79, 17.19)**
	**Unadjusted**		**Adjusted**	
	(n = 911; 70 fractures)		(n = 878; 67 fractures)	
	OR (95% CI)		OR (95% CI)	
**Fracture Status**				
Protein density				
Higher protein density	Ref		Ref	
Lower protein density	1.59 (0.97, 2.61)		**1.73 (1.02, 2.92)**	

Higher protein density = calories from protein ≥ 15%; Lower protein density = calories from protein < 15%; Injury status = all participants fell, outcome dichotomized into fell with and without any reported injury. A fall with injury was used as an event. Fracture status = all participants fell, outcome dichotomized into fell with and without any reported fracture. A fall with fracture was used as an event. Injury status and protein density had a significant interaction with diabetes medication use; therefore, only stratified analyses are present. As 13 subjects had missing data on diabetes medication use, they were excluded from stratified analyses. Adjusted for age at baseline, BMI category, physical activity (MET-hrs/wk), race/ethnicity, and Clinical trial arm. Abbreviations: BMI = Body mass index; MET-hrs/wk: the metabolic equivalent of task (hours/week); OR = Odds Ratio; CI = Confidence interval.

### Secondary analysis

Of the women with low protein by weight and who fell, 58 (35.8%) sought treatment for that injury, and 104 (64.2%) did not seek treatment. Participants with low protein by weight did not have a significant increase in odds of falling with a treated injury compared to those meeting their RDA of protein by weight (Table 4). Of the women with lower protein density who fell, 51 (36.4%) sought treatment for that injury, and 89 did not seek treatment (63.6%). HT use defined from WHI baseline (including medication use prior to WHI enrollment) to LLS enrollment was found to modify the effect between the association of protein density and treatment sought for injured falls (p = 0.01), so stratified models are presented. Table 4 shows the ORs for injury and lower protein density by HT use.

**Table 4:** Associations between protein by weight and protein density and treatment sought for injury status (n = 418) and protein by weight and protein density and injury with fracture status (n = 444) in older women in the Objective Physical Activity and Cardiovascular Health in Older Women (OPACH) study.

	**Unadjusted**		**Adjusted**	
	(n = 418; 149 treated)		(n = 407; 144 treated)	
	OR (95% CI)		OR (95% CI)	
**Treatment Sought for Injury**				
Protein by weight				
Normal protein	Ref		Ref	
Low protein	1.01 (0.67, 1.53)		0.88 (0.50, 1.58)	
	**Unadjusted**		**Adjusted**	
	**Never used HT**	**Ever used HT**	**Never used HT**	**Ever used HT**
	(n = 229; 76 treated)	(n = 189; 73 treated)	(n = 208; 68 treated)	(n = 176; 68 treated)
	OR (95% CI)	OR (95% CI)	OR (95% CI)	OR (95% CI)
**Treatment Sought for Injury**				
Protein density				
Higher protein density	Ref	Ref	Ref	Ref
Lower protein density	0.59 (0.31, 1.09)	1.83 (1.00, 3.36)	0.53 (0.27, 1.06)	1.85 (0.94, 3.63)
	**Unadjusted**		**Adjusted**	
	(n = 444; 70 fractures)		(n = 430; 67 fractures)	
	OR (95% CI)		OR (95% CI)	
**Injury with Fracture**				
Protein by weight				
Normal protein	Ref		Ref	
Low protein	1.27 (0.76, 2.13)		1.41 (0.67, 2.95)	
	**Unadjusted**		**Adjusted**	
	(n = 444; 70 fractures)		(n = 430; 67 fractures)	
	OR (95% CI)		OR (95% CI)	
**Injury with Fracture**				
Protein density				
Higher protein density	Ref		Ref	
Lower protein density	1.43 (0.85, 2.40)		1.46 (0.84, 2.53)	

Normal protein = weight * 0.8 ≤ total FFQ protein; Low protein = weight * 0.8 > total FFQ protein; Normal protein density = calories from protein ≥ 15%; Lower protein density = calories from protein < 15%; Treatment sought for injury = all participants fell with an injury and were dichotomized by whether they received treatment from a doctor or not. Treatment from a doctor was then the event. Injury with Fracture = all participants fell with an injury and were dichotomized into those who did and did not fall with a reported fracture. A fall with a reported fracture was the event. HT use was defined by all available HT use data from the WHI baseline to LLS enrollment, and participants were defined as never users if they had never indicated HT use, or ever users if they had ever indicated use. Treatment sought for injury status and protein density had a significant interaction with never using HT; therefore, only stratified analyses are present. Adjusted for age at baseline, BMI category, physical activity (MET-hrs/wk), race/ethnicity, and clinical trial arm membership. Protein by weight is additionally adjusted for total calories. Abbreviations: Ref = reference; OR = Odds Ratio; CI = Confidence interval; HT = hormone therapy; BMI = Body mass index; MET-hrs/wk = the metabolic equivalent of task (hours/week).

Among the women with low protein by weight, 144 (32.4%) reported an injury without fracture, and 31 (7.0%) reported an injury with fracture. Similarly, among women with lower protein density, 124 (27.9%) reported an injury without fracture, and 29 (6.5%) reported an injury with fracture. Both low protein by weight and protein density had no significant associations with injury with fracture risk (Table 4).

### Sensitivity analysis

In sensitivity analyses, we tested the associations between injury and fracture status and protein sources. In participants with lower protein density, their mean vegetable protein was 22.5 (12.2) g, whereas their mean animal protein was 29.7 g (Table 1). High vs low vegetable and animal proteins did statistically significantly differ between low and normal protein by weight (chi-square p^all^ < 0.001). High vs low vegetable and animal proteins did not differ between injury and fracture status (p^all^ > 0.2). There were no significant associations between animal and vegetable protein and injury from falling, or fracture from falling (Table 5).

**Table 5:** Associations between animal and vegetable protein intake and fall by injury status (n = 1285) and animal and vegetable protein and fracture from fall status (n = 911) in older women in the Objective Physical Activity and Cardiovascular Health in Older Women (OPACH) study.

	**Unadjusted**	**Adjusted**
	(n = 1285; 444 injured)	(n = 1241; 430 injured)
	OR (95% CI)	OR (95% CI)
**Injury Status**		
Animal protein (g)		
High animal protein	Ref	Ref
Low animal protein	0.87 (0.70, 1.10)	0.85 (0.67, 1.08)
Vegetable protein (g)		
High vegetable protein	Ref	Ref
Low vegetable protein	0.86 (0.68, 1.08)	0.85 (0.67, 1.08)
	**Unadjusted**	**Adjusted**
	(n = 911; 70 fractures)	(n = 878; 67 fractures)
	OR (95% CI)	OR (95% CI)
**Fracture Status**		
Animal protein (g)		
High animal protein	Ref	Ref
Low animal protein	1.07 (0.66, 1.75)	1.16 (0.68, 1.90)
Vegetable protein (g)		
High vegetable protein	Ref	Ref
Low vegetable protein	0.89 (0.55, 1.46)	0.86 (0.53, 1.47)

Animal protein cutoff = 37.72g; Vegetable protein cutoff = 21.08g; Injury status = all participants fell, outcome dichotomized into fell with and without any reported injury. A fall with injury was used as an event. Fracture status = all participants fell, outcome dichotomized into fell with and without any reported fracture. A fall with fracture was used as an event. Adjusted for age at baseline, BMI category, physical activity (MET-hrs/wk), race/ethnicity, and clinical trial arm membership. Abbreviations: BMI = Body mass index; MET-hrs/wk: the metabolic equivalent of task (hours/week); OR = Odds Ratio; CI = Confidence interval.

## Discussion

In summary, protein density (percent of calories from a protein source) was significantly associated with an increased risk of fall injury. Interestingly, there was a significant interaction with diabetes medication use and protein density associated with fall with injury (p = 0.01). Lower protein density in individuals who used diabetes medication was associated with an increased risk of falls with injury by 554% compared to those with higher protein density. Diabetes medication use, particularly insulin, has been linked to increased risk of falls and fall-related morbidity [28]. In the literature, associations between risk of falls and protein intake have been limited and indicate a possible beneficial association between high protein intake and risk of falls [9,11,15–17]. History of diabetes was only assessed as a covariate in one instance [16], and medication use usually consisted of any relevant medication if it was considered. Additionally, Zeraattalab-Motlagh et al. (2024) conducted a systematic review on the risk of protein and fracture risk [17]. Of the 20 papers included for review, only two examined history of diabetes as a covariate. Given the limited results involving diabetes and diabetes medication use, our study population may be novel compared to the populations in the literature, and replication is necessary. These findings argue that special consideration for diabetes medications, treated diabetes, and underlying conditions needs to be considered if a patient is at a higher risk of falls. However, due to the limited number of participants taking diabetes medications (n = 103) and the self-reported nature of the data, these findings must be interpreted with caution. Regardless, our results are in alignment in that protein intake is a potential modifiable risk factor in falls.

Additionally, lower protein density was significantly associated with an increased risk of falling with a fracture in the whole group compared to higher protein density. This association remained significant when we removed participants with self-reported fractures who did not report receiving medical care (S4 Table in S1 File). The metric for falling with an injury is important to understand, given the limited research on falling with any injury, but fractures are one of the most significant metrics of the severity of a fall to try to mitigate [1–3,17]. Fractures from falls alone are one of the highest healthcare system burdens in the world, and there are more fractures expected as one of the largest generations enters the at-risk group [1–3,17]. Low protein intake has been shown to be associated with fractures, poorer bone health, and frailty in the literature [6,7,12,16,17]. More specifically, in the WHI, it has been shown that low protein intake affects lean mass and physical function in older adults, which is strongly associated with fall risk [6,12,25]. These associations are at least partially related to the relationships between protein and skeletal muscle [17,29]. Increased protein is not only necessary for maintaining and increasing muscle mass, but also for bone health [6–8,10,12,16,17,29]. Much of the literature has noted that sarcopenia is a risk factor for risk of falls and fractures [1–3] and interestingly, grip strength and SPPB were not confounders using the more liberal 5% criterion in these associations. Our sample is limited to participants who fell, and there is no significant difference between falling with and without an injury based on grip strength (p-value = 0.90) and SPPB (p-value = 0.74). It is likely that limiting our sample to fallers removes the effect of physical function as all these participants have generally lowered function. Based on the literature and our findings, protein intake may further help mitigate the risk of falling with an injury or fracture. Replication of this study in a larger sample is necessary to better elucidate the associations with protein density and injurious falls and fractures among fallers.

In comparison, low animal, low vegetable, and low total protein by body weight were not associated with an increased risk of fall with injury or fall with a fracture. Studies have suggested that older adults need a higher protein intake, and that the RDA should be around 1.0–1.2 g per kg of weight [30]. In this study population, only 39.9% achieved 1.0g/kg weight of protein, whereas 59.8% achieved 0.8g/kg weight. For both of these cutoff points, there were no significant associations in this study. In the full WHI-FI study (n = 5,732), 56% of participants met the 0.8 g/kg RDA, indicating that this lower recommendation is achievable in older women and appears similar in our smaller sample [20]. The recent Dietary Guidelines for Americans (DGA) recommends 1.2–1.6 g/kg of protein compared to the 0.8 utilized in the RDA [31]. In this sample, 25.8% achieved the 1.2g/kg cutoff, and 7.8% achieved 1.6 g/kg. Though not shown in these analyses, using these new cutoff points did not yield significant findings between protein by weight and increased fall with injury, thus the RDA and DGA cutoff findings were in alignment.

The differences in significance based on protein measure are likely derived from how the measurement was created. The RDA for protein consumption was created without controlling for age, sex, and body composition, and is the minimum amount of protein needed to avoid losing body nitrogen [22,23]. Whereas, the AMDR was created to show dietary recommendations in the context of a complete diet [23]. As stated, the AMDR recommends that 10–35% of total calories should come from a protein source [22,23], and 15% has been previously used in the literature as the lower cutoff in healthy controls [13,22]. Animal and vegetable protein sources do not have separate standardized recommendations; therefore, we had to rely on median cutoff points, which may have masked an association at a lower threshold.

The participants in this study had overall lower energy intakes and poorer diet quality, and while the participants had fewer calories, most participants (67.9%) achieved at least 15% of their daily calories from protein compared to normal protein by weight (59.8%). Furthermore, in this group, 35.6% of participants had discordant pairs between protein density and protein by weight, and Cohen’s Kappa was 0.24. Women who met their RDA had an average total calorie intake of 1902.7 (±634.3) kcal compared to women above the 15% for protein density, who had 1,554.3 (±646.3) kcal. The HEI 2010 scores were 73.6 (±9.8) in the higher protein density group compared to the 72.7 (±10.2) in the normal protein by weight group. As protein density is intended as being a part of complete diet, it may better reflect dietary patterns and may potentially act as a diet balance proxy rather than a measurement of protein. In the whole group, total animal protein was nearly double (41.9g) compared to vegetable protein (22.8g). However, total vegetable protein was similar between low and normal protein groups, whereas there was a significant difference between low and normal protein groups in total animal protein. Additionally, only 37 (2.9%) women met their RDA with vegetable protein intake alone compared to 317 (24.7%) who met their RDA with only animal protein sources. These findings indicate that many of these women may have to change their diets to meet the protein by weight RDA by increasing their protein and/or total calorie intake. Furthermore, when compared to non-fallers with available protein data in LLS/FI/OPACH, the fallers were more likely to meet both their RDA and protein density goals (S5 Table in S1 File). Therefore, intervention work to improve protein intake in this population may be difficult and will require careful consideration of the protein metric best suited to the population and if an emphasis on complete diet quality may be preferable to individual macronutrients.

This study had several strengths, including using the LLS/FI/OPACH cohort. We prospectively followed a group for 13 months of daily falls surveillance who had already been a part of the WHI for at least 13 years [20,21]. This allowed for a comprehensive understanding of the participants prevalent comorbidities and falls history prior to data collection. The fall data were not from an annual fall questionnaire asking participants to do a one-year recall, but rather a daily calendar count. Interviews about the falls also occurred within two months of the fall [21]. All of these steps ensured a reduction in recall and information biases. However, this study did have some limitations. Due to limitations in study resources, OPACH was unable to capture fall injury information for every fall; therefore, only a participant’s first fall interview was included in the analysis. Given the associations with repeat falls and injury, more severe falls were likely missed in later follow-ups [11]. However, in avoiding these participants’ later interviews, we believe we have addressed the potential selection bias to the best of our ability. By using an FFQ with a 3-month reflection window, we have assumed that the single assessment represents a relatively stable dietary pattern over follow-up period, however, the possibility that dietary habits changed should be considered when interpreting results. Furthermore, several key findings came from stratified analyses with small samples. These findings must be carefully considered due to limited power and precision. Additionally, we were unable to assess potential residual confounding in the diabetes medication stratified models, specifically from chronic kidney disease (CKD) and diabetes severity. Those with diabetes often have CKD, and with CKD, there are restrictions in dietary protein consumption and an increase in frailty [32]. CKD prevalence and incidence were not assessed in the main WHI sample and the LLS/FI/OPACH cohort. A study with more detailed diabetes and kidney function data will be needed to assess these associations. Power was limited due to the smaller sample size, particularly in stratified analyses for treatment sought after injury analyses. Lastly, women in the LLS/FI/OPACH had been active study participants for at least 13 years, and the degree to which the findings can be generalized is unknown.

## Conclusions

Lower protein density was associated with increased risk of falling with a fracture and increased injury of any kind among older women who used diabetes medication. Adequate protein intake is critical for musculoskeletal health in older women, and both dietary and medication considerations should be considered for the prevention of injury outcomes from falls.

## Supporting information

S1 Table Injury by medical treatment in those who reported fall with an injury (n = 418) in older women in the Objective Physical Activity and Cardiovascular Health in Older Women (OPACH) study. Per WHI protocol, cells with less than 10 participants are reported as <10. Not all participants reported treatment, nor had all injury types; therefore, columns and rows do not equal 100%.
(DOCX)

S2 Table Fracture types in those who reported a fall with fracture (n = 70) in older women in the Objective Physical Activity and Cardiovascular Health in Older Women (OPACH) study. Lower limb included: Toe, Ankle, Knee, Leg, Thigh, and Hip; Upper Limb included: Finger, Arm, Wrist, and Shoulder; Other included: Back, Chest, Face, and Other; Per WHI protocol, cells with less than 10 participants are reported as <10, therefore only combined body areas are presented as no specific fracture area had more than 10 cases.
(DOCX)

S3 Table Associations between protein by weight (1.0g protein cutoff) with injury from fall and injury with fracture in older women in the Objective Physical Activity and Cardiovascular Health in Older Women (OPACH) study. Normal protein = weight * 1.0 ≤ total FFQ protein; Low protein = weight * 1.0 > total FFQ protein; Injury status = all participants fell, outcome dichotomized into fell with and without any reported injury. A fall with injury was used as an event. Fracture status = all participants fell, outcome dichotomized into fell with and without any reported fracture. A fall with fracture was used as an event. Adjusted for total calories, age at baseline, BMI category, physical activity (MET-hrs/wk), race/ethnicity, and clinical trial arm membership. Abbreviations: BMI = Body mass index; MET-hrs/wk: the metabolic equivalent of task (hours/week); OR = Odds Ratio; CI = Confidence interval.
(DOCX)

S4 Table Associations between protein by weight and protein density and fracture from fall status and injury with fracture in older women in the Objective Physical Activity and Cardiovascular Health in Older Women (OPACH) study, with confirmed medical treatment for fracture. Normal protein = weight * 0.8 ≤ total FFQ protein; Low protein = weight * 0.8 > total FFQ protein; Normal protein density = calories from protein ≥ 15%; Lower protein density = calories from protein < 15%; Fracture status = all participants fell, outcome dichotomized into fell with and without any reported fracture. A fall with fracture was used as an event. Injury with Fracture = all participants fell with an injury and were dichotomized into those who did and did not fall with a reported fracture. A fall with a reported fracture was the event. Adjusted for age at baseline, BMI category, physical activity (MET-hrs/wk), race/ethnicity, and clinical trial arm membership. Protein by weight is additionally adjusted for total calories. Abbreviations: BMI = Body mass index; MET-hrs/wk: the metabolic equivalent of task (hours/week); OR = Odds Ratio; CI = Confidence interval.
(DOCX)

S5 Table Descriptive statistics between study sample of women who fell (n = 1285) and non-fallers from the Objective Physical Activity and Cardiovascular Health in Older Women (OPACH) study with available FFQ data (n = 2776). Non-fallers included all participants from OPACH who were only excluded for not falling. Abbreviations: BMI = Body mass index; MET-hrs/wk: the metabolic equivalent of task (hours/week).
(DOCX)
