# Supplementary material for: Protein intake and injury outcomes among fallers in the Women’s Health Initiative’s Objective Physical Activity and Cardiovascular Health in Older Women Study
Source: PLoS One. 2026 Jul 22;21(7):e0353769. doi: 10.1371/journal.pone.0353769 (PMC13390837; doi:10.1371/journal.pone.0353769)
Supplement: S1 Table — Per WHI protocol, cells with less than 10 participants are reported as <10. Not all participants reported treatment, nor had all injury types; therefore, columns and rows do not equal 100%. (DOCX) [file pone.0353769.s001.docx]

**Supplemental Table S1**: Injury by medical treatment in those who reported fall with an injury (n=418) in older women in the Objective Physical Activity and Cardiovascular Health in Older Women (OPACH) study

|  | Self-reported Injury | | | | | |
| --- | --- | --- | --- | --- | --- | --- |
| Medical Treatment for Fall with Injury | Sore  N (%) | Brusing  N (%) | Scrape  N (%) | Cut  N (%) | Sprain  N (%) | Fracture  N (%) |
| Treated the injury by yourself | 196 (46.9) | 186 (44.5) | 104 (24.9) | 28 (6.7) | 26 (6.2) | 10 (2.4) |
| Treated by someone who was not a doctor | 19 (4.5) | 15 (3.6) | 10 (2.4) | 10 (2.4) | <10 | <10 |
| Went to Doctors office | 46 (11.7) | 32 (7.7) | 12 (2.9) | <10 | 17 (4.1) | <10 |
| Went to Emergency Room | 59 (14.1) | 49 (11.7) | 25 (6.0) | 23 (5.5) | 19 (4.5) | 31 (7.4) |
| Admitted to hospital | 11 (2.6) | <10 | <10 | <10 | <10 | 13 (3.1) |
| Per WHI protocol, cells with less than 10 participants are reported as <10. Not all participants reported treatment, nor had all injury types; therefore, columns and rows do not equal 100% | | | | | | |
