# Supplementary material for: Protein intake and injury outcomes among fallers in the Women’s Health Initiative’s Objective Physical Activity and Cardiovascular Health in Older Women Study
Source: PLoS One. 2026 Jul 22;21(7):e0353769. doi: 10.1371/journal.pone.0353769 (PMC13390837; doi:10.1371/journal.pone.0353769)
Supplement: S2 Table — Lower limb included: Toe, Ankle, Knee, Leg, Thigh, and Hip; Upper Limb included: Finger, Arm, Wrist, and Shoulder; Other included: Back, Chest, Face, and Other; Per WHI protocol, cells with less than 10 participants are reported as <10, therefore only combined body areas are presented as no specific fracture area had more than 10 cases. (DOCX) [file pone.0353769.s002.docx]

**Supplemental Table S2:** Fracture types in those who reported a fall with fracture (n=70) in older women in the Objective Physical Activity and Cardiovascular Health in Older Women (OPACH) study

| Fracture | N (%) |
| --- | --- |
| Lower Limb | 28 (40.0) |
| Upper Limb | 26 (37.1) |
| Other | 16 (22.9) |
| Lower limb included: Toe, Ankle, Knee, Leg, Thigh, and Hip; Upper Limb included: Finger, Arm, Wrist, and Shoulder; Other included: Back, Chest, Face, and Other; Per WHI protocol, cells with less than 10 participants are reported as <10, therefore only combined body areas are presented as no specific fracture area had more than 10 cases | |
