# Supplementary material for: Protein intake and injury outcomes among fallers in the Women’s Health Initiative’s Objective Physical Activity and Cardiovascular Health in Older Women Study
Source: PLoS One. 2026 Jul 22;21(7):e0353769. doi: 10.1371/journal.pone.0353769 (PMC13390837; doi:10.1371/journal.pone.0353769)
Supplement: S3 Table — Normal protein = weight * 1.0 ≤ total FFQ protein; Low protein = weight * 1.0 > total FFQ protein; Injury status = all participants fell, outcome dichotomized into fell with and without any reported injury. A fall with injury was used as an event. Fracture status = all participants fell, outcome dichotomized into fell with and without any reported fracture. A fall with fracture was used as an event. Adjusted for total calories, age at baseline, BMI category, physical activity (MET-hrs/wk), race/ethnicity, and clinical trial arm membership. Abbreviations: BMI = Body mass index; MET-hrs/wk: the metabolic equivalent of task (hours/week); OR = Odds Ratio; CI = Confidence interval. (DOCX) [file pone.0353769.s003.docx]

**Supplemental Table S3:** Associations between protein by weight (1.0g protein cutoff) with injury from fall and injury with fracture in older women in the Objective Physical Activity and Cardiovascular Health in Older Women (OPACH) study

|  | **Unadjusted** | **Adjusted** |
| --- | --- | --- |
|  | (n=1285; 444 injured) | (n=1241; 430 injured) |
| **Injury Status** | OR (95% CI) | OR (95% CI) |
| Protein by weight |  |  |
| Normal protein | Ref | Ref |
| Low protein | 1.03 (0.82, 1.31) | 1.24 (0.89, 1.72) |
|  | **Unadjusted** | **Adjusted** |
|  | (n=911; 70 fractures) | (n=878; 67 fractures) |
| **Fracture Status** | OR (95% CI) | OR (95% CI) |
| Protein by weight |  |  |
| Normal protein | Ref | Ref |
| Low protein | 1.21 (0.73, 2.01) | 1.53 (0.75, 3.11) |
| Normal protein = weight * 1.0 ≤ total FFQ protein; Low protein = weight * 1.0 > total FFQ protein; Injury status = all participants fell, outcome dichotomized into fell with and without any reported injury. A fall with injury was used as an event. Fracture status = all participants fell, outcome dichotomized into fell with and without any reported fracture. A fall with fracture was used as an event. Adjusted for total calories, age at baseline, BMI category, physical activity (MET-hrs/wk), race/ethnicity, and clinical trial arm membership. Abbreviations: BMI = Body mass index; MET-hrs/wk: the metabolic equivalent of task (hours/week); OR = Odds Ratio; CI = Confidence interval | | |
