# Supplementary material for: Protein intake and injury outcomes among fallers in the Women’s Health Initiative’s Objective Physical Activity and Cardiovascular Health in Older Women Study
Source: PLoS One. 2026 Jul 22;21(7):e0353769. doi: 10.1371/journal.pone.0353769 (PMC13390837; doi:10.1371/journal.pone.0353769)
Supplement: S4 Table — Normal protein = weight * 0.8 ≤ total FFQ protein; Low protein = weight * 0.8 > total FFQ protein; Normal protein density = calories from protein ≥ 15%; Lower protein density = calories from protein < 15%; Fracture status = all participants fell, outcome dichotomized into fell with and without any reported fracture. A fall with fracture was used as an event. Injury with Fracture = all participants fell with an injury and were dichotomized into those who did and did not fall with a reported fracture. A fall with a reported fracture was the event. Adjusted for age at baseline, BMI category, physical activity (MET-hrs/wk), race/ethnicity, and clinical trial arm membership. Protein by weight is additionally adjusted for total calories. Abbreviations: BMI = Body mass index; MET-hrs/wk: the metabolic equivalent of task (hours/week); OR = Odds Ratio; CI = Confidence interval. (DOCX) [file pone.0353769.s004.docx]

**Supplemental Table S4:** Associations between protein by weight and protein density and fracture from fall status and injury with fracture in older women in the Objective Physical Activity and Cardiovascular Health in Older Women (OPACH) study, with confirmed medical treatment for fracture

|  | **Unadjusted** | **Adjusted** |
| --- | --- | --- |
|  | (n=929; 57 fractures) | (n=896; 55 fractures) |
| **Fracture Status** | OR (95% CI) | OR (95% CI) |
| Protein by weight |  |  |
| Normal protein | Ref | Ref |
| Low protein | 1.30 (0.75, 2.27) | 1.63 (0.75, 3.53) |
| Protein density |  |  |
| Normal protein density | Ref | Ref |
| Lower protein density | 1.72 (0.98, 3.02) | **1.98 (1.09, 3.57)** |
| **Injury with Fracture** | **Unadjusted**  (n=439; 57 fractures)  OR (95% CI) | **Adjusted**  (n=426; 55 fractures)  OR (95% CI) |
| Protein by weight |  |  |
| Normal protein | Ref | Ref |
| Low protein | 1.43 (0.80, 2.54) | 1.71 (0.74, 3.93) |
| Protein density |  |  |
| Normal protein density | Ref | Ref |
| Lower protein density | 1.55 (0.86, 2.77) | 1.64 (0.89, 3.03) |
| Normal protein = weight * 0.8 ≤ total FFQ protein; Low protein = weight * 0.8 > total FFQ protein; Normal protein density = calories from protein ≥ 15%; Lower protein density = calories from protein < 15%; Fracture status = all participants fell, outcome dichotomized into fell with and without any reported fracture. A fall with fracture was used as an event. Injury with Fracture = all participants fell with an injury and were dichotomized into those who did and did not fall with a reported fracture. A fall with a reported fracture was the event. Adjusted for age at baseline, BMI category, physical activity (MET-hrs/wk), race/ethnicity, and clinical trial arm membership. Protein by weight is additionally adjusted for total calories. Abbreviations: BMI = Body mass index; MET-hrs/wk: the metabolic equivalent of task (hours/week); OR = Odds Ratio; CI = Confidence interval | | |
