# Supplementary material for: Protein intake and injury outcomes among fallers in the Women’s Health Initiative’s Objective Physical Activity and Cardiovascular Health in Older Women Study
Source: PLoS One. 2026 Jul 22;21(7):e0353769. doi: 10.1371/journal.pone.0353769 (PMC13390837; doi:10.1371/journal.pone.0353769)
Supplement: S5 Table — Non-fallers included all participants from OPACH who were only excluded for not falling. Abbreviations: BMI = Body mass index; MET-hrs/wk: the metabolic equivalent of task (hours/week). (DOCX) [file pone.0353769.s005.docx]

**Supplemental Table S5:** Descriptive statistics between study sample of women who fell (n=1285) and non-fallers from the Objective Physical Activity and Cardiovascular Health in Older Women (OPACH) study with available FFQ data (n=2776)

|  | **Study Sample** | **Non-fallers** |
| --- | --- | --- |
|  | (N=1,285) | (n=2,776) |
| **Variables** | N (%) / Mean (SD) | N (%) / Mean (SD) |
| Age at OPACH baseline (yrs) | 79.2 (6.5) | 77.8 (6.7) |
| Race & Ethnicity |  |  |
| White | 801 (62.4%) | 1715 (63.1%) |
| Black/African American | 278 (21.7%) | 938 (34.5%) |
| Hispanic/Latina | 183 (14.3%) | 500 (18.0%) |
| BMI (kg/m^2^) | 26.6 (5.3) | 27.6 (5.7) |
| BMI (kg/m^2^)* |  |  |
| Normal weight (BMI 18.5-24.9) | 526 (41.2%) | 917 (33.2%) |
| Overweight (BMI 25.0-29.9) | 428 (33.5%) | 1003 (36.4%) |
| Obese (BMI ≥30.0) | 293 (23.0%) | 786 (28.5%) |
| Physical Activity (MET-hrs/wk) | 14.3 (15.0) | 12.8 (15.1) |
| Total calories (kcal) | 1565.3 (681.8) | 1480.8 (695.5) |
| Total Protein (g) | 64.7 (29.8) | 60.7 (29.4) |
| Low Protein by Weight | 768 (59.8%) | 1360 (49.0%) |
| Lower Protein Density | 873 (67.9%) | 922 (33.2%) |
| Non-fallers included all participants from OPACH who were only excluded for not falling. Abbreviations: BMI = Body mass index; MET-hrs/wk: the metabolic equivalent of task (hours/week) | | |
